# The third Symptom Management Research Trial in Oncology (SMaRT Oncology-3): a randomised trial to determine the efficacy of adding a complex intervention for major depressive disorder (*Depression Care for People with Lung Cancer*) to usual care, compared to usual care alone in patients with lung cancer

**DOI:** 10.1186/1745-6215-10-92

**Published:** 2009-09-30

**Authors:** Jane Walker, Jim Cassidy, Michael Sharpe, SMaRT Oncology-3 Trialists

**Affiliations:** 1 Cancer Research UK, The University of Edinburgh Cancer Research Centre, Western General Hospital, Crewe Road, Edinburgh, UK; 2 Cancer Research UK, Department of Medical Oncology, University of Glasgow, Garscube Estate, Switchback Road, Glasgow, UK; 3 Psychological Medicine Research, School of Molecular and Clinical Medicine, the University of Edinburgh, Kennedy Tower, Royal Edinburgh Hospital, Morningside Park, Edinburgh, UK

## Abstract

**Background:**

*Depression Care for People with Lung Cancer *is a complex intervention delivered by specially trained cancer nurses, under the supervision of a psychiatrist. It is given as a supplement to the usual care for depression, which patients receive from their general practitioner and cancer service. The third Symptom Management Research Trial in Oncology (SMaRT Oncology-3 Trial) will test its efficacy when compared to usual care alone.

**Design:**

A two arm parallel group multi-centre randomised controlled trial. 200 patients will be recruited through established systematic Symptom Monitoring Services, which screen patients for depression. Patients will have: a diagnosis of lung cancer; an estimated life expectancy of three months or more and a diagnosis of Major Depressive Disorder. Patients will be randomised to usual care or usual care plus *Depression Care for People with Lung Cancer*. Randomisation will be carried out by telephoning a secure computerised central randomisation system or by using a secure web interface. The primary outcome measure is average depression severity. This will be assessed using scores on the 20-item Symptom Hopkins Checklist (SCL-20D), collected every four weeks over 32 weeks. Secondary outcomes include severity of anxiety, pain and fatigue; self-rated improvement of depression; quality of life and satisfaction with depression care.

**Trial Registration:**

Current controlled trials ISRCTN75905964

## Background

Depression is a major problem in patients with lung cancer. A large survey of cancer patients found that those with lung cancer had the highest rate of psychological distress [1]. In addition, depression has been reported in one third of patients with inoperable lung cancer on entry to clinical trials, with persistence of depressive symptoms in half of these patients [2].

A systematic review of psychosocial interventions for patients with lung cancer found only three small randomised controlled trials [3]. The interventions tested in these, and subsequent trials, have reported modest benefits on depressive symptoms, but none have selected depressed patients or evaluated interventions intended to treat depression. There is, therefore, very little evidence to guide the practical management of depression in patients with lung cancer.

We have developed a complex intervention (*Depression Care for People with Cancer*) for depression in cancer patients with good prognosis. The SMaRT Oncology-1 Trial found that patients who received the intervention had significantly better outcomes than those who received usual care alone at three months and that this difference was sustained at six and twelve months [4]. Whilst these results are encouraging, they cannot be generalised to patients with lung cancer, who have a relatively limited life expectancy and different needs. We, therefore, propose to evaluate the efficacy of an adapted form of *Depression Care for People with Cancer *in patients with lung cancer (*Depression Care for People with Lung Cancer*).

### Trial hypotheses

Supplementing usual care with *Depression Care for People with Lung Cancer *will improve the following over eight months (32 weeks):

• depressive symptoms

• other symptoms (pain, fatigue, anxiety)

• functioning

• quality of life

• satisfaction with depression care

## Methods

### Design

A two-arm parallel group randomised controlled trial with outcome data collection every 4 weeks to 32 weeks post-randomisation.

### Patients

200 patients will be recruited from lung cancer outpatient clinics in NHS Scotland. The majority of patients will be recruited from cancer clinics in NHS Lothian and NHS Greater Glasgow and Clyde.

To be included in the trial patients must:

• Have a diagnosis of lung cancer.

• Be aged 18 or over.

• Have a predicted survival, estimated by their cancer specialist, of three months or more.

• Have symptoms which meet DSMIV criteria for Major Depressive Disorder (MDD), with symptoms of the current Major Depressive Episode (MDE) present for four weeks or more using the inclusive approach to diagnosis [5].

Patients will be excluded if:

• They are unable to provide informed consent to participate.

• The episode of depression is chronic (defined as a history of continuous depression for at least two years).

• They are judged to require urgent psychiatric care.

• They are receiving active psychiatric or psychological treatment from specialist mental health services.

• They have cognitive impairment or communication difficulties (including inability to adequately understand verbal explanations or written information in English) which are incompatible with the intervention.

• They have known cerebral metastases.

• They are unable to participate regularly in treatment sessions.

• The intervention is judged to be inappropriate due to a medical condition which requires alternative treatment.

• The intervention is judged to be inappropriate due to a psychiatric condition which requires alternative treatment (psychotic illness, bipolar affective disorder, obsessive compulsive disorder, substance abuse or dependence).

• Their participation in the trial is judged to be inappropriate on other clinical grounds.

N.B Patients receiving active cancer treatments will not be excluded unless they fulfil one or more of the exclusion criteria listed above.

### Patient identification and enrolment

In order to obtain a representative sample, the majority of patients will be identified through the Symptom Monitoring Service (SMS) in outpatient clinics attended by lung cancer patients. The SMS is part of the routine NHS care for patients attending these clinics. It comprises two stages:

Stage 1: Patients complete self-report questionnaires in the clinic to evaluate their symptoms, including emotional distress (Hospital Anxiety and Depression Scale, HADS). A summary of each patient's symptoms is generated for their oncology team.

Stage 2: Patients with high emotional distress scores (HADS total score of 15 or more) receive a brief telephone-delivered, depression screening interview. When clinically relevant, a report is generated for the patient's general practitioner (GP) and oncologist.

The SMS staff will ask patients, with an MDE of at least four weeks' duration and a cancer prognosis of three months or more, to give permission for their contact details to be passed to the research team. It will be made clear that patients who do so will not be obliged to participate in the trial. Clinicians may wish to refer patients to the trial from other lung cancer clinics. In this instance, the patient will be referred to Stage 2 of the SMS.

Once patients have given permission, the research team will provide them with a trial information leaflet (usually by post) and will contact them directly (usually by telephone). Patients will be informed, during the telephone call, that if they are interested in taking part, their suitability for inclusion in the trial must first be assessed. Information will be required from patients' medical records in order to achieve this.

Patients will therefore be asked, at this stage, to give verbal consent for the research team to access their medical records. This will minimise the number of unnecessary face-to-face assessments. Consent to access medical records is required as the researchers do not provide the patient's usual clinical care. The patient's records will be used to determine the presence or absence of specific inclusion and exclusion criteria, before they are seen for a face-to-face assessment (if appropriate). If the patient does not give consent for the research team to access their records at this stage, consent will be obtained during the face-to-face assessment.

At the face-to-face assessment, patients will be given a full explanation of the two treatment allocations and the procedures for randomisation and outcome data collection. Written informed consent will then be obtained from patients for the eligibility assessment and, if appropriate, trial participation. Patients will be asked to consent to the information gathered about them being retained even if they are ineligible to participate in the trial. This information will be used to determine the generalisability of the trial findings.

The information retained from medical records and the face-to-face assessment will be used to determine eligibility. Eligibility assessments will be carried out by trained research staff (senior nurses and psychiatrists) and will include administration of the major depression component of the Structured Clinical Interview for DSMIV (SCID) to confirm the patient's diagnosis of major depression. If the patient is eligible to participate, their willingness to do so will be confirmed before randomisation. The patient's details will be entered into a database via an automated telephone service or secure website. The patient's treatment allocation will be automatically generated. The research team will inform the patient of their allocation either face-to-face or by telephone. At all stages the research team will endeavour to record reasons for non-participation.

### Trial treatment - comparison (usual care)

The patient's GP and oncologist will be informed of their diagnosis of MDD. Patients will receive the usual clinical management for their depression. Data will be collected from all patients to allow a retrospective description of the 'usual care' that they receive.

### Trial treatment - intervention (usual care supplemented with *Depression Care for People with Lung Cancer*)

In addition to the usual care described above, patients will receive a complex intervention, *Depression Care for People with Lung Cancer*. The intervention will be delivered by specially trained Care Managers (cancer nurses) under the supervision of the SMaRT Psychiatry team. Patients will be allocated to Care Managers systematically, being allocated to the next Care Manager on the appropriate site-specific list. If the allocated Care Manager is unable to see the patient for their first treatment session within two weeks (e.g. due to leave or staff sickness), the patient will be allocated to the next listed Care Manager.

The intervention is described in detail in the *Depression Care for People with Lung Cancer Treatment Manual *and comprises two phases:

a) Treatment Phase: patients will be offered a maximum of ten 30-45 minute sessions with their Care Manager over a 16 week period (patients are expected to receive an average of six to eight sessions).

b) Maintenance phase: patients will then receive active treatment follow-up by telephone every four weeks until the end of their participation in the trial.

During the Treatment Phase the patient's Care Manager will: (a) Coordinate their depression care by liaising with all relevant health professionals; (b) Monitor their symptoms of depression, using a brief standardised depression questionnaire (Patient Health Questionnaire-9, PHQ-9), at each session; (c) Provide a brief psychological intervention comprising education about depression and its management (including the use of antidepressant medication, being active and coping with problems better) and Problem Solving Treatment. A member of the SMaRT Psychiatry Team will attend a session, along with the Care Manager, early in the Treatment Phase, when it is necessary to advise the patient's GP regarding the prescription of antidepressant medication. Treatment sessions will be delivered at the cancer clinic, at home, in the hospice.

During the Maintenance Phase patients will be asked to complete the PHQ-9 at monthly follow-up telephone calls. Patients' responses to this questionnaire will be reviewed by their Care Manager and appropriate action will be taken as necessary.

All patients will be discussed during weekly supervision sessions, provided by the SMaRT Psychiatry Team. In addition, a member of the Psychiatry Team will be available to respond to emergencies. At any stage of treatment patients who report suicidal thoughts will be discussed with the Psychiatry Team and an appropriate management plan will be implemented. A supervising psychiatrist will review all patients who in their opinion: (a) require a diagnostic re-assessment; (b) have failed to achieve a treatment response (defined as a 50% drop in their PHQ-9 score *and *a PHQ-9 score of <10) by session five or week eight of the Treatment Phase (whichever is earlier); (c) require assessment for referral to general adult psychiatric care (patients with high suicide risk, psychosis or mania and those requiring inpatient care).

Patients' adherence to the intervention will be monitored by recording their attendance at treatment sessions, completion of homework, antidepressant prescription (drug and dose) and self-reported compliance with medication.

In order to ensure Care Manager's compliance with the treatment manual all treatment sessions will be video/audio recorded with the patient's permission.

Recordings and treatment notes will be reviewed regularly by the SMaRT Psychiatry Team and a randomly selected 10% sample of recordings will be independently reviewed to assess adherence to the Treatment Manual, rated on a standardised adherence checklist, designed specifically for this intervention.

### Primary outcome measure

The primary outcome measure is average depression severity. This will be assessed using SCL-20D scores, collected every four weeks over 32 weeks.

### Secondary outcome measures

The following secondary outcome measures are listed below. These will also be assessed using an average of the scores collected every four weeks over 32 weeks:

• Patients' self-rated improvement of depression, measured by a five point global improvement scale.

• Severity of anxiety symptoms, measured by the SCL-10A.

• Severity of pain and fatigue, measured by the relevant symptom scales of the EORTC QLQ-C30.

• Physical, social and role functioning, and overall health and quality of life measured by the relevant scales of the EORTC QLQ-C30.

• Patient's satisfaction with depression care, measured by a 5-point Likert scale item developed specifically for the trial.

### Measures of satisfaction with *Depression Care for People with Lung Cancer*

Assessed at 16 weeks, in patients randomised to usual care supplemented with *Depression Care for People with Lung Cancer *using a semi-structured interview.

### Measures of cost and health-related quality of life

The following economic outcome measures will be assessed at 4, 8, 12, 16, 20, 24, 28 and 32 weeks from randomisation:

• Cost of 'usual care' treatments received for depression.

• Cost of health care service use (primary, secondary and community based).

• Cost of the trial intervention.

• Health related quality of life, measured by EQ-5D.

### Data collection

Baseline data will be obtained by the assessor at the end of the face-to-face assessment as near as possible prior to randomisation.

Outcome data will be obtained by telephone or by face-to-face interview by a dedicated team who are blind to group allocation and are situated and managed independently from the trial team. This team will be managed by the Scottish Mental Health Research Network (SMHRN). Outcome data will be collected at 4, 8, 12, 16, 20, 24, 28 and 32 weeks from randomisation. The date of completion of all questionnaires will be noted. Data collection will aim to be within one week either side of the specified follow-up date. Measures of satisfaction with the trial intervention will be collected by a researcher separate from the outcome data collection team.

### Data collection instruments

The following data will be collected from patients at baseline, 4, 8, 12, 16, 20, 24, 28 and 32 weeks:

• Symptom Checklist Depression Scale (SCL-20D).

• Symptom Checklist Anxiety Scale (SCL-10A).

• European Organisation for Research and Treatment of Cancer Quality of Life Questionnaire (EORTC-QLQ-C30).

• Patient's satisfaction with depression care (question developed specifically for the trial).

• EuroQol-5D (EQ-5D).

• Treatments received for depression (measures developed specifically for the trial).

• Use of health care services (measures developed specifically for the trial).

At baseline, demographic details and information about the patient's cancer will also be collected. Satisfaction with the trial intervention will be assessed at 16 weeks using a semi-structured interview, developed specifically for the trial.

### Sample size

The sample size of 200 will give 90% power at the 5% significance level to detect a standardised difference of 0.46, that is a mean difference of 46% of the underlying standard deviation. It is anticipated that this standard deviation will be of the order of 0.6 to 0.7. The trial will have 80% power to detect a standardised difference of 0.40.

### Method of randomisation

In order to ensure similarity in the baseline characteristics of the two treatment groups randomisation will be carried out using a combination of stratification and minimisation. Stratification will be by trial centre (Edinburgh, Glasgow) and minimisation variables will be age (≤60, 61-69, ≥70), lung cancer type (small cell, non-small cell, other) and sex. The process of stratification and minimisation will be carried out by a telephone/web-based randomisation system developed by the Division of Clinical Neuroscience (DCN) Clinical Trials Unit at the University of Edinburgh.

### Statistical analyses

A single main analysis will be performed at the end of the trial when all outcome data have been collected. An independent Data Monitoring Committee will review confidential interim analyses of accumulating data at least annually. A detailed Statistical Analysis Plan will be developed prior to closure of the trial database and prior to the unblinding of the treatment allocations.

The main analysis will be performed blind to treatment allocation on the 'intention to treat' principle. With such an ill patient population it is anticipated that missing values will be common, either resulting from individual missed assessments or due to patients dying or deteriorating to such an extent that they become unable to comply with assessments. This will be handled in the primary analysis by using an 'area under the curve' approach to calculate the mean SCL-20D score. The areas will be calculated using the trapezoidal rule.

Recognising that this is an efficacy trial, a 'per protocol' analysis will also be performed and reported. Patients will be excluded from the per protocol analysis if they were allocated to 'usual care plus Depression Care for People with Lung Cancer' and received fewer than four treatment sessions. This criterion will be applied without knowledge of outcome.

### Economic analyses

The economic analyses will examine the cost-effectiveness of the complex intervention from an NHS perspective. Resource usage will be collected in the trial in relation to primary health care, community health and secondary health care services. These data will be collected from questionnaires administered to patients at each follow up point. The questionnaires will be designed for this study and will be based on questionnaires used in economic evaluations of other interventions.

Unit cost estimates, from routine published sources such as PSSRU Unit Costs of Health and Social Care [6], will then be applied to resource use data collected above to generate patient level cost estimates which will be presented as a mean cost with a measure of uncertainty. EQ-5D data will facilitate estimation of health-related quality of life, which will be expressed in terms of health states within the 245-state classification and in terms of health state values based on the preferences of a sample of the UK public [7]. Mean utility for each treatment allocation will be estimated with a measure of uncertainty. The analyses above will enable the estimation of mean differences in costs and effects for the treatment and control allocations. These will be presented with 95% confidence intervals around the differences in costs and effects. Uncertainty will be represented using the cost effectiveness acceptability curve (CEAC), a graphical representation of the probability of an intervention being cost-effective [8].

### Approvals and sponsorship

SMaRT Oncology-3 has been approved, with favourable assessments at all sites, by the Scotland A Research Ethics Committee (ref: 08/MRE00/95). Management approval has been granted by the Research and Development Offices of NHS Lothian and NHS Greater Glasgow. The trial is co-sponsored by the University of Edinburgh and NHS Lothian Health Board.

### Timescales

Recruitment commenced January 2009. The target for recruitment closure is November 2010, with outcome data collection continuing until July 2011.

## Competing interests

The authors declare that they have no competing interests.

## Authors' contributions

JW participated in the design and coordination of the trial and drafted the manuscript. JC participated in the coordination of the trial. MS conceived of the trial, participated in its design and coordination and helped to draft the manuscript. All authors read and approved the final manuscript.
